# Bovine Lactoferrin Decreases Cholera-Toxin-Induced Intestinal Fluid Accumulation in Mice by Ganglioside Interaction

**DOI:** 10.1371/journal.pone.0059253

**Published:** 2013-04-08

**Authors:** Fulton P. Rivera, Anicia M. Medina, Sandra Bezada, Roberto Valencia, María Bernal, Rina Meza, Ryan C. Maves, Theresa J. Ochoa

**Affiliations:** 1 Instituto de Medicina Tropical Alexander von Humboldt, Universidad Peruana Cayetano Heredia, Lima, Peru; 2 Laboratorio de Fisiopatogenia, Departamento de Fisiología, Facultad de Medicina, Universidad de Buenos Aires, Buenos Aires, Argentina; 3 Laboratorio de Farmacología, Facultad de Farmacia y Bioquímica, Universidad Nacional Mayor de San Marcos, Lima, Peru; 4 Facultad de Veterinaria y Zootecnia, Universidad Peruana Cayetano Heredia, Lima, Peru; 5 Department of Bacteriology, U.S. Naval Medical Research Unit SIX, Lima, Peru; 6 Division of Infectious Diseases, Naval Medical Center San Diego, San Diego, California, United States of America; 7 Center for Infectious Diseases, University of Texas School of Public Health, Houston, Texas, United States of America; University of North Dakota, United States of America

## Abstract

Secretory diarrhea caused by cholera toxin (CT) is initiated by binding of CT’s B subunit (CTB) to GM1-ganglioside on the surface of intestinal cells. Lactoferrin, a breast milk glycoprotein, has shown protective effect against several enteropathogens. The aims of this study were to determine the effect of bovine-lactoferrin (bLF) on CT-induced intestinal fluid accumulation in mice, and the interaction between bLF and CT/CTB with the GM1-ganglioside receptor. Fluid accumulation induced by CT was evaluated in the mouse ileal loop model using 56 BALB/c mice, with and without bLF added before, after or at the same time of CT administration. The effect of bLF in the interaction of CT and CTB with GM1-ganglioside was evaluated by a GM1-enzyme-linked immunosorbent assay. bLF decreased CT-induced fluid accumulation in the ileal loop of mice. The greatest effect was when bLF was added before CT (median, 0.066 vs. 0.166 g/cm, with and without bLF respectively, p<0.01). We conclude that bLF decreases binding of CT and CTB to GM1-ganglioside, suggesting that bLF suppresses CT-induced fluid accumulation by blocking the binding of CTB to GM1-ganglioside. bLF may be effective as adjunctive therapy for treatment of cholera diarrhea.

## Introduction

Diarrheal disease due to enteric infection is a major cause of death among children under five years of age, especially in developing countries. Enterotoxigenic bacteria are responsible for a large proportion of these diseases. Among the enterotoxin-producing bacteria, *Vibrio cholerae* causes the most severe disease, while enterotoxigenic *Escherichia coli* (ETEC) is responsible for the largest number cases. ETEC is also a major cause of diarrhea in adult travelers from industrialized countries to the developing world. The major virulence factors of these bacteria are their homologous enterotoxins, cholera toxin (CT) and heat-labile enterotoxin (LT), respectively. These toxins are examples of bacterial AB_5_ toxins, consisting of one enzymatically active A subunit (CTA or LTA) that assemble with five B subunits (CTB or LTB) which are responsible for the toxins’ binding properties [1].

Breastfeeding has been identified as one of the most effective interventions to prevent pediatric diarrhea as well as all-cause mortality [2]. Lactoferrin is a major nonimmune milk factor that has been thought to be important in protecting infants from intestinal infections. It is an iron-binding 78-kDa glycoprotein that is resistant to proteolytic enzymes [3], [4], [5], [6]. Lactoferrin is produced not only in breast milk but also in other mucosal secretions and phagocytic cells. Both human and bovine lactoferrin may protect against Gram-negative bacteria in a variety of ways [7]. It was originally thought that it impaired bacterial multiplication due to its ability to decrease availability of iron required for growth [8], [9]. However, the antibacterial activity of lactoferrin is not due solely to its bacteriostatic iron-binding capacity [10]. A pepsin-derived fragment of lactoferrin has iron independent bactericidal activity that is associated with release of lipopolysaccharide (LPS). However, undigested human and bovine lactoferrin are able to interact with LPS thus inducing bactericidal effect due to release of LPS [11], [12], [13]. Lactoferrin kills or slows bacterial growth synergistically with other factors that may be present in mucosal secretions, including immunoglobulins, complement, and lysozyme [7].

The mechanism of the diarrhea induced by both CT and LT is initiated by the binding of B subunits to the GM1-ganglioside present on the surface of intestinal epithelial cells [14], [15]. This binding induces a conformational change in the toxin, followed by the translocation of the A subunit into the enterocyte. Inside these cells, CTA and LTA catalyze the ADP-ribosylation of the stimulatory GTP-binding protein, resulting in increased intracellular levels of cyclic AMP. Elevated levels of cyclic AMP in the cells cause massive loss of fluid and electrolytes from the cell, eventually resulting in diarrhea [14]. Since the interaction between toxin and receptor is the first step of CT- and LT-induced diarrhea, the binding of CTB and/or LTB to GM1 is an attractive target for developing drugs for the treatment and prevention of CT- and LT-induced diarrhea [15], [16]. The aims of this study were to determine the effect of bovine lactoferrin (bLF) on the cholera-toxin-induced intestinal fluid accumulation in mice, and on the interaction between bLF and CT or CTB with the GM1-ganglioside receptor. We also tested the effect of bLF on interaction between *in vitro E. coli*-produced LT and GM1-ganglioside receptor.

## Results

### Bovine Lactoferrin Inhibits ETEC H10407 Growth

**Figure 1:**
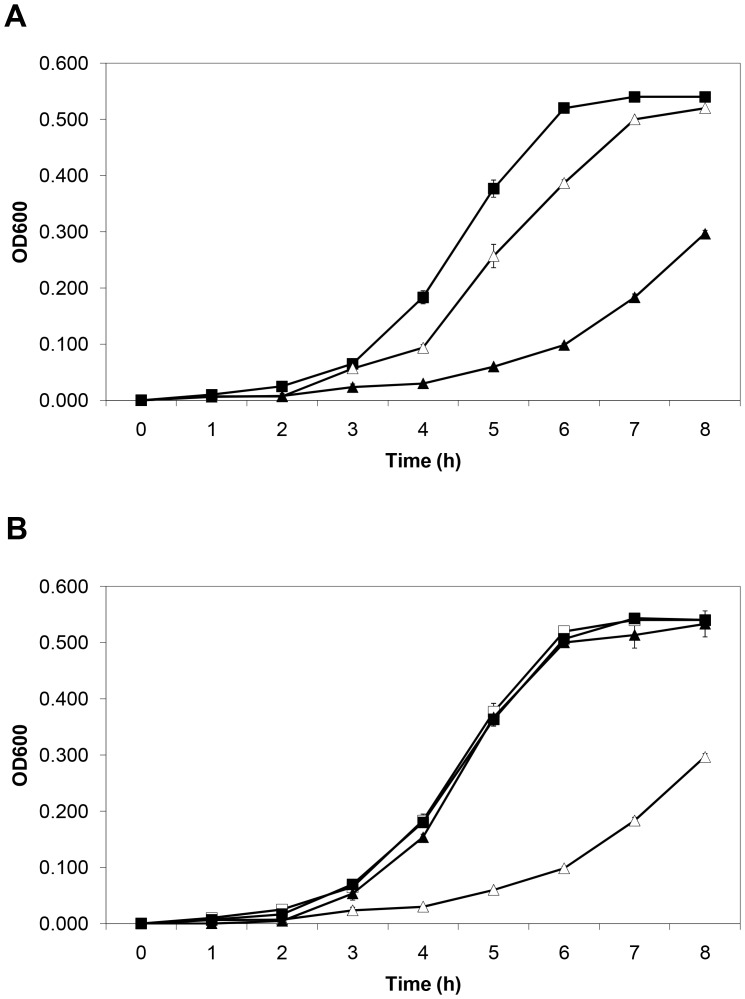
Effect of bovine lactoferrin concentrations on ETEC H10407 growth. A) Effect of two lactoferrin concentrations on ETEC H10407 growth. Bacteria were incubated on Luria Broth (control) (▪) or bovine lactoferrin at 1.0 mg/mL (Δ) or 10.0 mg/mL (▴) for 8 h. Bacterial growth was monitored spectrophotometrically at OD600 for 8 h. B) Effect of iron and lactoferrin on ETEC growth. Bacteria were incubated in Luria Broth (control) with (▪) or without iron (□), or bovine lactoferrin at 10 mg/mL with (▴) and without iron (Δ) (4∶1 molar ratio of iron to lactoferrin). Bacterial growth was monitored spectrophotometrically at OD600 for 8 h (mean ± SD of 3 experiments). The SD bars are not seen in the majority of the samples because they fall inside the symbol.

**Figure 2:**
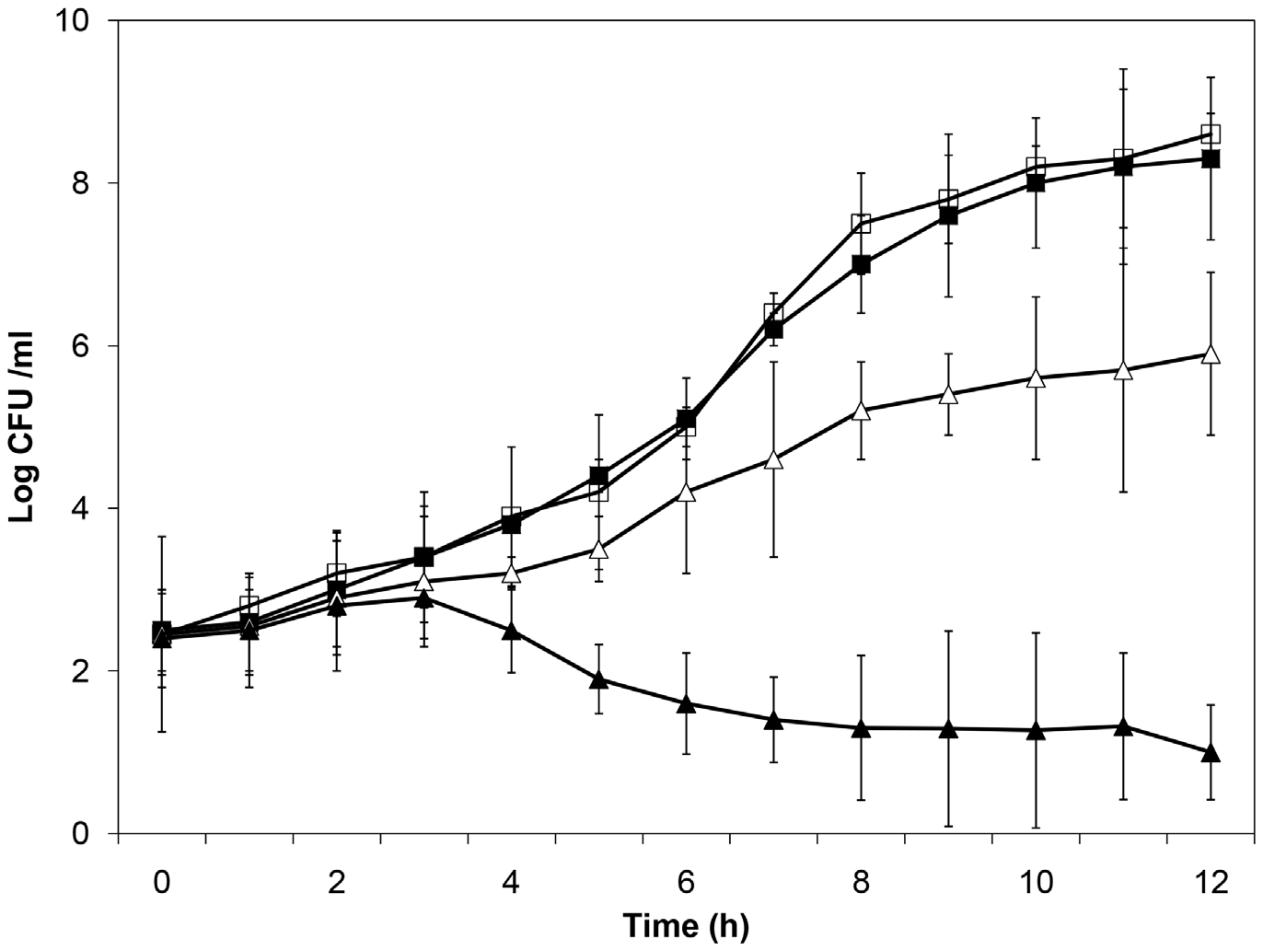
Effect of lactoferrin concentrations on ETEC H10407 growth with and without an excess of ferric chloride. Bacteria were incubated on Luria Broth (control) (▪) or bovine lactoferrin at 1.0 mg/mL (Δ) or 10.0 mg/mL (▴) or bovine lactoferrin at 10 mg/mL with an excess of ferric chloride (□) (4∶1 molar ratio of iron to lactoferrin) for 12 h. Three dilutions of the bacterial culture were plated on agar to determine the number of colonization forming units (CFU) (mean ± SD of 3 experiments).

Bovine lactoferrin inhibited ETEC H10407 growth in a dose-dependent manner (Figure 1A). During logarithmic growth phase (5–7 h), there was a significant difference between the slopes of the growth curves of the control and bLF (10 mg/mL) groups (*p*<.001). Also, there was significant growth inhibition with bLF at a concentration of 1 mg/mL (*p*<.001) at 5 and 6 h during logarithmic growth phase. It is known that lactoferrin inhibits the growth of many pathogenic bacteria because of its iron-binding capacity [9]. The inhibitory effect of bLF on H10407 was iron-dependent, with iron reversing the growth inhibition (Figure 1B). We next evaluated whether this effect on growth was bacteriostatic or bacteriocidal. ETEC were grown in LB broth with or without bLF at a concentration of 10 mg/mL for 6 hours. At this time, the bacteria pellets were washed twice with PBS, resuspended in LB broth without bLF, and incubated for an additional 4 hours. Bacteria whose growth was significantly inhibited by bLF (OD600 0.098 vs 0.520, bLF vs control, respectively) had normal growth when put into media without bLF (OD600 0.570 vs 0.545, lactoferrin vs control, respectively). Thus, bLF inhibited ETEC H10407 growth without impairing viability. Likewise, we determined by the plate count that there was a significant difference between the slopes of the growth curves of the control and bLF (10 mg/mL) groups during the logarithmic growth phase (5–12 h) (*p*<.001) (Figure 2). Also, there was significant growth inhibition with bLF at a concentration of 1 mg/mL (*p*<.001) at 8–10 h during logarithmic growth phase. Similar to the results of the OD measurement assay, the inhibitory effect of bLF on H10407 was iron-dependent, with iron reversing the growth inhibition (Figure 2).

### bLF Inhibits the Binding of *E. coli*-produced LT to GM1

**Figure 3:**
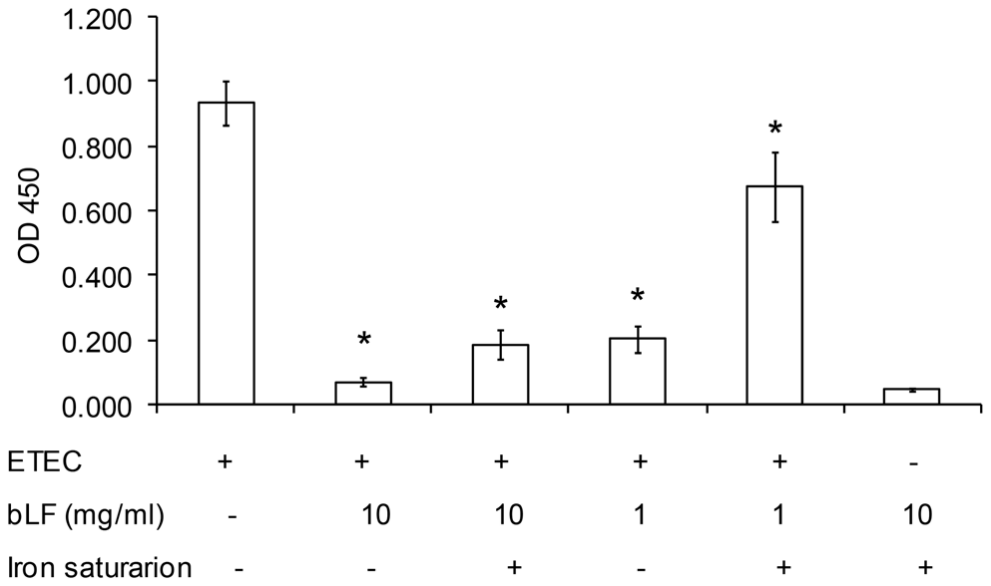
Inhibitory ability of bovine lactoferrin (bLF) on the binding of LT produced by ETEC H10407 to GM1 by GM1-ELISA. The wells were coated with 500 ng of GM1 and ETEC H10407 was incubated on LB broth with 100 µL of bLF (10 mg/mL or 1 mg/mL) with and without iron (4∶1 molar ratio of iron to lactoferrin) and incubated at 37°C overnight. The absorbance was read at 450 nm in an ELISA plate reader. Values are mean ± standard desviation of three independent assays. *, *p*<.001 for the comparison of bLF groups with and without iron saturation vs. LT produced by ETEC group, by unpaired t test.

We analyzed the effect of bLF on the binding of *in vivo E. coli*-produced LT to GM1 by competitive GM1-ELISA. As shown in Figure 3, bLF inhibited the binding of LT to GM1 in a concentration-dependent manner. The inhibitory ability was higher in bLF concentration of 10 mg/ml (93.4%) vs. concentration of 1 mg/ml (81.1%), *p*<.001. When iron saturated lactoferrin was used, the inhibitory ability decreased up to 82.6% and 36.8%, in 10 mg/ml and 1 mg/ml bLF groups, respectively.

### bLF Inhibits the CT-induced Fluid Accumulation in Mice

**Figure 4:**
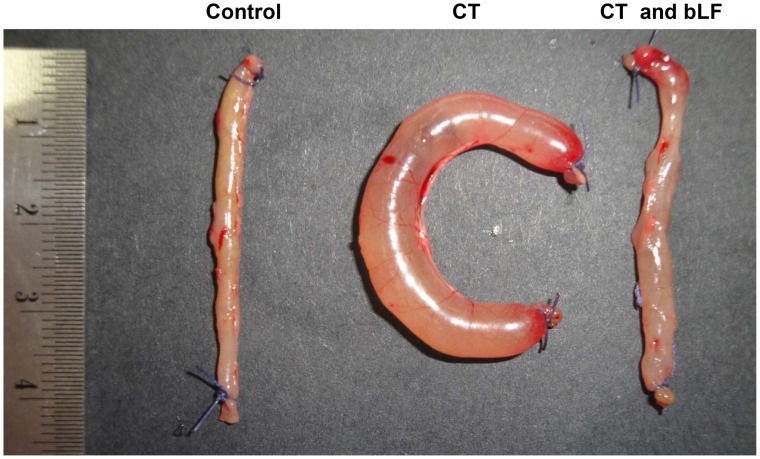
Inhibitory effect of bovine lactoferrin (bLF) on CT-induced fluid accumulation in the mouse ileal loop. Control (phosphate-buffered saline, pH7.4) or CT (1.5 µg) without or with bLF (10 mg/mL) were simultaneously injected into the ileal loops, and mice were sacrificed 24 h later.

**Figure 5:**
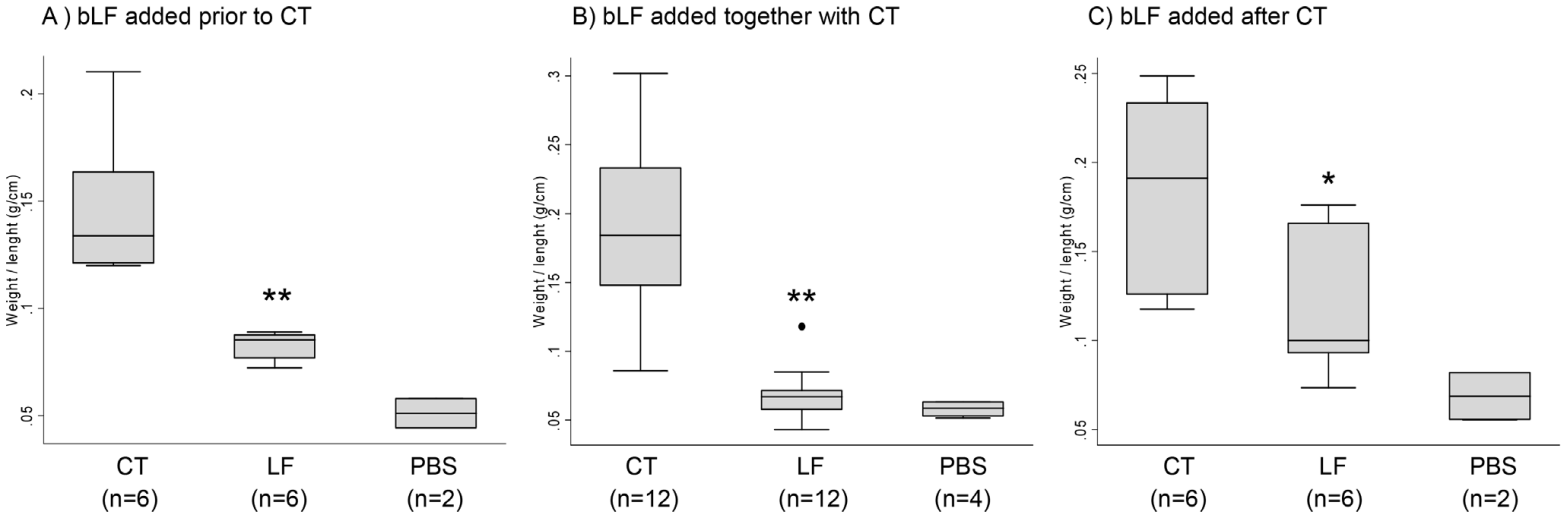
Effect of bovine lactoferrin (bLF) on CT-induced fluid accumulation in ileal loops. CT (1.5 µg) was added to the mouse ileal loop with bLF (10 mg/mL). Weigh/length ratios were calculated. A) bLF added before CT. Each loop was injected with 0.1 mL of sample solution (phosphate-buffered saline [PBS], 2 mg/loop bLF, PBS control) and 10 min later CT (1.5 µg) was added, B) bLF added together or at the same time with CT, and C) bLF added 10 min after CT. *, *p*<.05; **, *p*<.01 for the comparison of bLF group vs. CT groups, by Mann–Whitney U test.

Since one of the biological activities of CT is the induction of fluid accumulation in the intestine, we analyzed the *in vivo* antidiarrheal efficacy of bLF using the toxin-induced fluid accumulation assay in the short circuit of small intestines in mice. In this assay, the closed loops of small intestines were created *in vivo*, and the lumens of loops were injected with small volumes of CT, CT plus bLF or PBS (control). Luminal fluid accumulation was determined after 24 h. As seen in Figure 4, there was marked fluid accumulation and distention in CT-treated loops, whereas the normal (PBS) loop remained empty. Also, the bLF significantly suppressed fluid accumulation in the toxin-treated intestinal loops when it was added before or after cholera toxin (Figure 5). PBS alone did not affect the fluid accumulation induced by CT.

### bLF Inhibits the CT-induced Fluid Accumulation by Blocking the Binding of CTB to GM1

**Figure 6:**
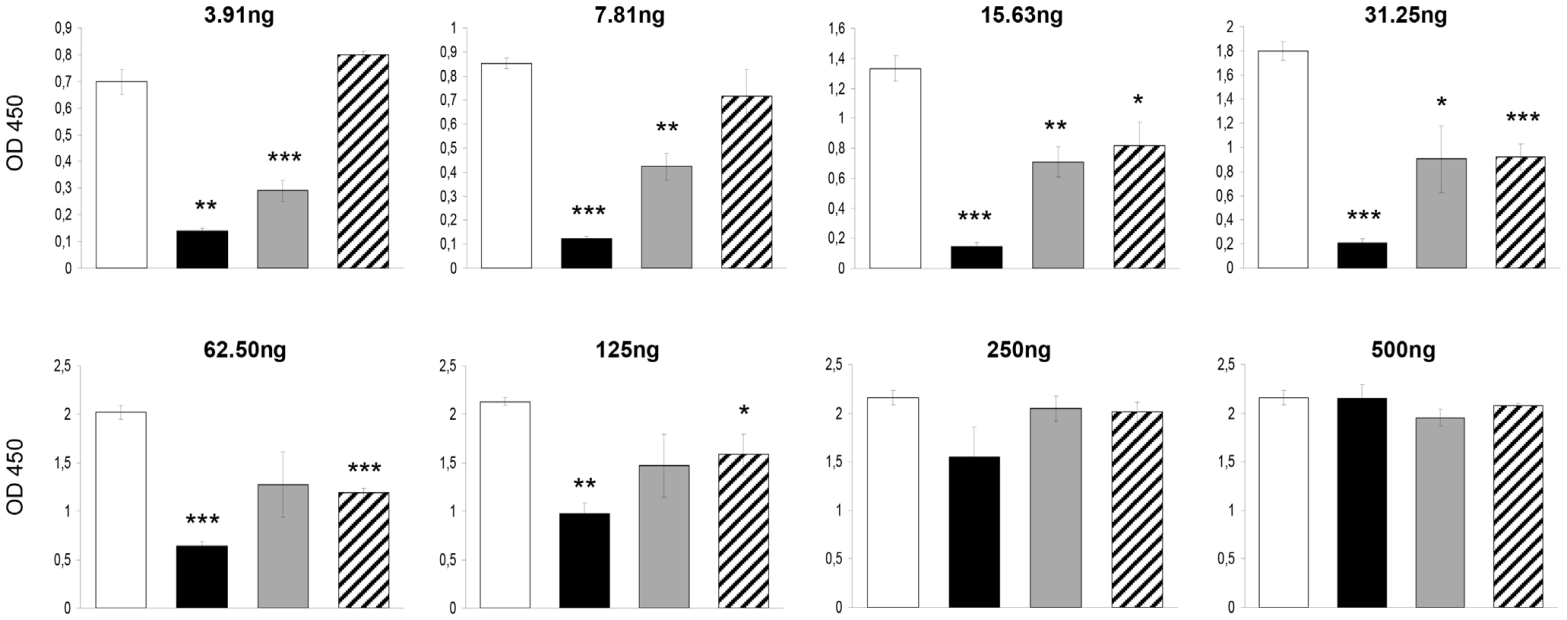
Inhibitory ability of bovine lactoferrin (bLF) on the binding of CT (gradient of concentration) to GM1 by GM1-ELISA. CT was added at a two fold increase in doses (3.91–500 ng) after (black bars), together (grey bars) or before (striped boxes) the bovine lactoferrin administration, and incubated in total for 3 h. The white bars represent the control group (CT alone). The absorbance was read at 450 nm in an ELISA plate reader. Values are mean ± standard desviation of three independent assays. *, *p*<.05; **, *p*<.01; ***, *p*<.001 for the comparison of bLF groups vs. CT group (CT alone), by unpaired t test.

**Figure 7:**
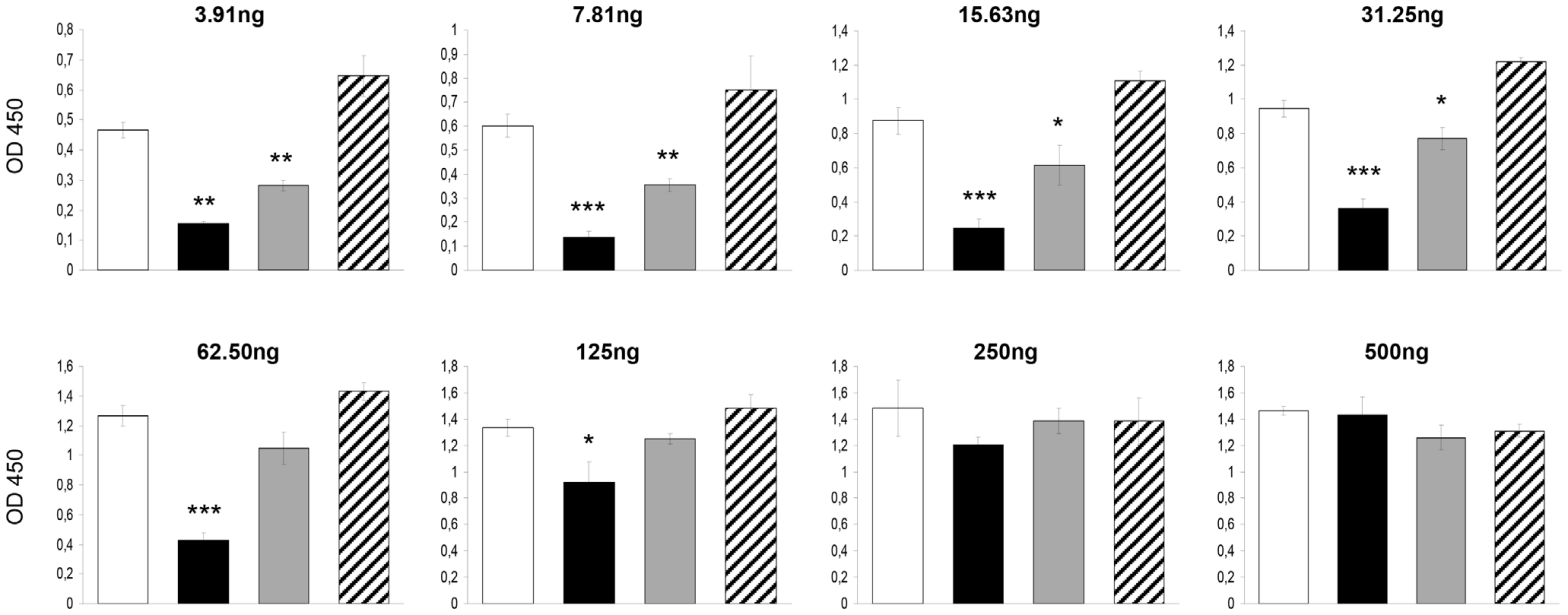
Inhibitory ability of bovine lactoferrin (bLF) on the binding of CTB (gradient of concentration) to GM1 by GM1-ELISA. CTB was added at a two fold increase in doses (3.91–500 ng) after (black bars), together (grey bars) or before (striped boxes) the bovine lactoferrin administration, and incubated in total for 3 h. The white bars represent the control group (CTB alone). The absorbance was read at 450 nm in an ELISA plate reader. Values are mean ± standard desviation of three independent assays. *, *p*<.05; **, *p*<.01; ***, *p*<.001 for the comparison of bLF groups vs. CT group (CT alone), by unpaired t test.

The inhibitory ability of bLF on the binding of cholera-toxin or the CT´s B subunit to GM1 was evaluated by competitive GM1-ELISA. bLF was mixed with various concentrations up to 500 ng of CT or CTB, incubated at room temperature for 3 hours, and added to GM1-coated wells. We also tested the effect the bLF when it was added before or after the CT or CTB. bLF significantly inhibited the binding of CT to GM1 (Figure 6). bLF was more effective when added before or together with CT; it was less effective when added after CT (Figure 6). The highest effect was when bLF was added before CT (OD mean ± SD of 3 experiments, 0.210±0.036) vs. the control group (CT alone, 31.25 ng) (1.797±0.081), *p*<.001. Similar findings were obtained with bLF and CTB. The highest effect was when bLF was added before CTB (OD mean ± SD of 3 experiments, 0.424±0.051) vs. the control group (CT alone, 62.5 ng) (1.266±0.072), *p*<.001. However, in this case there was no inhibitory effect when bLF was added after CT administration (Figure 7). The inhibitory effect of bLF differs depending on CT- or CTB-concentration with higher inhibitory capacity with CT or CTB concentration below 125 ng per well. Therefore, these findings suggest that the bLF blocks the binding of CTB to GM1, resulting in the suppression of CT-induced fluid accumulation.

## Discussion

There is currently no specific prophylaxis or therapy against diarrhea caused by bacterial enterotoxins. Oral rehydration salt (ORS) solution is a commonly used fluid/salt replacement therapy for infectious diarrhea. Although the ORS solution effectively aids recovery from dehydration, the combination of ORS solution with pharmacological agents that suppress the severe diarrhea by inactivating CT and/or LT would be advantageous. Antibacterial drugs may kill bacteria but cannot directly inhibit bacterial toxins once produced. Moreover, antibacterial drug therapy is limited by the rapid increase in drug resistance among bacteria, particularly in endemic areas [17]. Antimotility agents like loperamide can lessen stool frequency and volume in mild diarrheas. However, such agents are unsuitable in severe diarrhea because they might cause pooling of large fluid volumes in paralyzed bowel loops [18].

The discovery of agents that can block the function of toxin could be potentially important in the prevention and treatment of bacterial toxin-induced diarrhea. We screened the antidiarrheal potential of bLF on the basis of the CTB–GM1 interaction. Its antidiarrheal abilities were further evaluated by a fluid accumulation assay. Our results demonstrate a correlation between the inhibition of the CTB–GM1 interaction and the suppression of CT-induced diarrhea. Kawasaki *et al* previously reported that bLF and glycomacropeptide effectively reduced the CT-derived morphological changes in CHO-K1 cells. They reported that bLF inhibited the binding of CT to GM1. The inhibitory effect of lactoferrin seemed to be attributed binding to the terminal sialic acid, although the sugar chain sequence only partially fitted to the receptor [19]. By GM1-ELISA, we found that bLF blocks the binding of LT produced by *E. coli,* with a higher inhibitory effect on the CT-GM1 interaction when was added simultaneously or prior to CT or CTB. Therefore, bLF might interact with LTB or CTB to block their binding to GM1. Additionally, because the binding of toxin to GM1 is the first step for the translocation of A subunit into cells, blocking the toxin and GM1 interaction may abort subsequent events, such as the ADP-ribosylation of G protein and the elevation of cyclic AMP. Because LT and CT are two closely related toxins, these data suggested that bLF might not only inhibit CT-induced diarrhea but also inhibit LT-induced diarrhea.

It is well-recognized that lactoferrin has antimicrobial activity against many different pathogens including intestinal bacterias [20]. Lactoferrin has a bactericidal effect on ETEC strains isolated from pigs *in vitro* [21]. Binding of lactoferrin to *E. coli* colonization factors [22] causes inhibition of hemagglutination [23] and inhibition of adherence of ETEC to epithelial cells *in vitro* [24] and to intestinal mucosa of germfree mice *in vivo* [25]. Therefore, we speculated that bLF might exhibit the anti-diarrheal ability by both the suppression of intestinal bacterial growth and the blocking of binding of toxin to cellular receptor.

Studies of bLF supplementation have been conducted in infants to determine its effect on fecal flora and iron status [7], [26]. A previous study conducted in Peruvian children with acute watery diarrhea and dehydration showed that adding recombinant human lactoferrin and lysozyme to oral rehydration solution reduced the duration and recurrence of diarrhea [27].

Cholera is the most severe of the diarrheal diseases, with fluid losses of up to 30 liters per day. Indeed, without appropriate electrolyte and fluid replacement therapy, up to 30% of cholera patients can die from dehydration. Under-reporting of cholera is a notorious problem, and the often-cited figures of 3 million to 5 million cases and 120,000–200,000 deaths due to cholera annually could represent a small proportion, perhaps only 10–20% of all cases [28], [29]. Cholera remains today an important disease in areas where population overcrowding and poor sanitation are common, such as in slums and refugee camps in developing countries [30]. On the other hand, enteric infections caused by ETEC, are the most common cause of diarrhea in the developing world, being responsible for a total of as many as 1 billion cases yearly, of which 300–400 million arise in children under five years of age [31]. ETEC is also the most common cause of travelers’ diarrhea, being responsible for one-third to one-half of all diarrheal episodes in travelers to Africa, Asia and Latin America. Although the illness is typically mild, it results in an estimated 300,000–500,000 deaths per year, mostly in young children [28].

Vaccination may be the best avenue whereby we can eventually gain control over cholera. However, the highest protection level (85%) by the currently-available oral cholera vaccine generally lasts only six months. More long-lasting protection is desirable, and this could require additional antigens and/or improved immunization protocols. Research in this area is important, especially because of its high impact on public health in poor areas [32]. Taking into account the new trends in the epidemiology of cholera, as well as the changes in the evidence of both the economic burden of the disease and the effectiveness and feasibility of available cholera vaccines, new strategies concerning cholera prevention should be considered and implemented.

This study has some limitations. First, we have conducted the “iron-saturated” experiments using a mixed carried out with a 4∶1 molar ratio of iron to lactoferrin; however, this methodology cannot exclude the presence of free iron. Therefore, all the reported results on iron-saturated bLF activity and conclusion could not be assumed to be identical to those of holo-lactoferrin (lactoferrin iron-saturated at 100%). The experiments with holo-bLF should be made using bLF completely iron saturated (100%) obtained by adding adequate molar ratios of ferric ions and removing unbound ferric ions i.e. by Ajello *et al* methodology [33]. Additionally, we have not evaluated the effects of differing bLF concentrations on the fluid accumulation assay. Future studies should be carried out to determine these effects.

In this study, we demonstrated that bLF, a major non-immune milk factor, was effective in inhibiting cholera-induced fluid accumulation in mice via the abolishment of toxin–receptor interaction. Further studies of bovine lactoferrin are warranted for the prevention and treatment of cholera and ETEC-associated diarrhea in developing countries.

## Materials and Methods

### Lactoferrin

bLF was obtained from Tatua Biologics (Tatua, New Zealand). The purity of the LF was 90% with an iron saturation of 15%. bLF was used at two concentrations: 10.0 and 1.0 mg/mL. The concentrations of 10 mg/mL (0.125 mmol/L) and 1 mg/mL (0.0125 mmol/L) correspond approximately to the concentrations of lactoferrin present in human colostrum and mature milk, respectively.

### Bacterial Strain

*Escherichia coli* O78:H11 strain H10407 (CFA/I: LT+: ST+), a well-characterized virulent strain was used throughout this study. ETEC H10407 was cultivated overnight on CFA agar plates (1% amino acids, 0.15% yeast extract, 2% agar, 0.04 mm MnCl_2_ and 0.4 mm MgSO_4_, 0.15% bile salts).

### Growth Measurement of ETEC H10407 Strain

Following overnight growth on CFA plates, bacteria were inoculated in Luria-Bertani (LB) broth at a pH of 7.4, with or without lactoferrin, and incubated at 37°C. ETEC were also grown in LB broth in the presence of bovine lactoferrin with and without an excess of ferric chloride (4∶1 molar ratio of iron to lactoferrin). Bacterial growth was monitored spectrophotometrically at OD600 every hour for 8 h. Additionally, samples were taken at selected times over 12 h and serially diluted with sterile LB broth, and viable counts were determined by the plate count. The LB medium does not contain Fe as an ingredient, and no special precautions were taken to minimize Fe from media and cultures.

### GM1-Enzyme-Linked Immunosorbent Assay (GM1-ELISA)

GM1-ELISA to detect LT (produced *in vitro* by ETEC H10407) was performed as previously described [34], with some modifications. ELISA microtiter wells were coated with GM1 (0.5 µg/mL in phosphate-buffered saline [PBS] at room temperature overnight), and the ETEC H10407 strain was grown in 100 µL of LB broth with and without bLF (10 mg/mL or 1 mg/mL), with and without iron saturation, and supplemented with lincomycin (45 µg/mL) and glucose (2.5 mg/mL) in GM1-coated microtiter wells at 37°C overnight. Released LT from the ETEC H10407 strain was bound to the solid-phase GM1 and detected by means of an anti-LT monoclonal antibody (MAb) incubated for 90 minutes at room temperature. Detection of bound MAbs was performed using goat anti-mouse immunoglobulin G (IgG0 horseradish peroxidase followed by incubation for 90 minutes. Finally, H_2_O_2_ and ortho-phenylenediamine were added as substrates in order to visualize the results. The absorbance was read at 450 nm in an ELISA plate reader. The bLF inhibitory ability (%) was calculated by [1−(OD value of mixture containing released LT and bLF/OD value of mixture containing released LT alone)]×100.

### Fluid Accumulation Assay

Experimental animals were female mice BALB/c strain between 6 and 8 weeks of age with a weight between 20 and 24 g. Mouse experiments were conducted following ethical review and approval by the Institutional Animal Care and Use Committees of Universidad Peruana Cayetano Heredia (OLAW Assurance A5146-01) and Naval Medical Research Unit SIX (NAMRU-6, OLAW Assurance A4312-01), both in Lima, Peru, in strict accordance with the recommendations in the Guide for the Care and Use of Laboratory Animals of the National Institutes of Health. All procedures were performed under anesthesia with ketamine–acepromazine–xylazine (KAX), and all efforts were made to minimize suffering. Animals were healthy prior to inoculation.

Fluid accumulation induced by cholera toxin (Sigma-Aldrich, St. Louis, MO, USA) was evaluated with mouse ileal loops as described previously [35]. Briefly, mice were fasted for 24 h with water available ad libitum. Mice were then anesthetized with KAX, and the intestines were exteriorized through a midline incision. One intestinal segment (about 4 cm) was ligated, and the CT (1.5 µg) with or without lactoferrin (2 mg) in a total volume of 200 µL or only PBS (control) were simultaneously injected into the loop. Additionally, we tested the effect of bLF added before or after to cholera toxin. Briefly, each loop was injected with 100 µL of sample solution (2 mg bLF/loop or PBS) and 10 min later CT was added (1.5 µg in 100 µL); or each loop was injected with 100 µL of sample solution (1.5 µg CT/loop) and 10 min later bLF or PBS were added. Twenty-four hours later, the mice were sacrificed and the loops were excised. The fluid accumulation (g/cm) was calculated by dividing the weight of the fluid-containing loop by the length of the loop.

### Competitive Binding Assay

The interaction of cholera toxin (CT) and cholera toxin’s B subunit (CTB) (Sigma-Aldrich, St. Louis, MO, USA) with ganglioside GM1 was evaluated by the GM1-ELISA as described above. We tested the effect of bLF (10 mg/mL) added together either to CT or CTB in concentrations from 3.91–500 ng in a final volume of 200 µL. Wells coated with GM1 were incubated with 200 µL of CT or CTB plus bLF or PBS (control) for 3 hours at room temperature with shaking. For comparing the assay with the animal experiment, we also tested the effect of bLF added prior or after to CT or CTB. GM1-coated wells were incubated with 100 µL of bLF (20 mg/mL) for 1.5 hours at room temperature with shaking and 1.5 h after CT, CTB or PBS was added; or wells were incubated with 100 µL of CT or CTB for 1.5 hours at room temperature with shaking and 1.5 hours after bLF or PBS was added respectively. The absorbance was read as described above.

### Statistical Analysis

The results were analyzed using Stata, version 10.1 (Stata). Chi-square or Fisheŕs exact tests were used for comparisons between groups as appropriate. Student’s *t*-test for normally distributed data, or the Mann-Whitney U test for non-normally distributed data were used to compare continuous variables where appropriate. Significance was defined as a *p*<.05 for all analyses.
